# Absorption of Nickel, Chromium, and Iron by the Root Surface of Primary Molars Covered with Stainless Steel Crowns

**DOI:** 10.1155/2010/326124

**Published:** 2011-01-17

**Authors:** David Keinan, Eliyahu Mass, Uri Zilberman

**Affiliations:** ^1^Laboratory of Bioanthropology and Ancient DNA, School of Dental Medicine, The Hebrew University of Jerusalem, P.O. Box 12272, Jerusalem 91120, Israel; ^2^Clinic of Pediatric Dentistry, Barzilai Medical Center, 2nd Hahistadrut Street, Ashkelon 78278, Israel

## Abstract

*Objective*. The purpose of this study was to analyze the absorption of metal ions released from stainless steel crowns by root surface of primary molars. *Study Design*. Laboratory research: The study included 34 primary molars, exfoliated or extracted during routine dental treatment. 17 molars were covered with stainless-steel crowns for more than two years and compared to 17 intact primary molars. Chemical content of the mesial or distal root surface, 1 mm apically to the crown or the cemento-enamel junction (CEJ), was analyzed. An energy dispersive X-ray spectrometer (EDS) was used for chemical analysis. *Results*. Higher amounts of nickel, chromium, and iron (5-6 times) were found in the cementum of molars covered with stainless-steel crowns compared to intact molars. The differences between groups were highly significant (*P* < .001). *Significance*. Stainless-steel crowns release nickel, chromium, and iron in oral environment, and the ions are absorbed by the primary molars roots. The additional burden of allergenic metals should be reduced if possible.

## 1. Introduction

Nickel sensitivity is common and increasing in prevalence. Nickel was named the “contact allergen of the year” in 2008 by the American Contact Dermatitis Society (ACDS) because of its significant public health importance [1]. Nickel has been the most frequently detected allergen in patch-test populations worldwide, and in North America the prevalence of nickel sensitivity has been increasing steadily since the mid-1980s [2]. Contact dermatitis to nickel can significantly limit an individual's lifestyle, and allergy to nickel has health implications because of the use of nickel in implanted medical devices and in dentistry. 

Nickel, an abundant natural element, is a hard, silvery-white material in its pure state, which can be combined with other metals, for example, iron, copper, chromium, and zinc, to form alloys and stainless steel. All soil contains nickel and it is accumulated in plants. It is found in meteorites and on the ocean floor and is emitted from volcanoes. Small amounts of nickel are naturally found in drinking water and food. Smokers have a high nickel uptake through their lungs. In humans, most ingested and unabsorbed nickel is excreted in the feces [3]. Nickel absorbed from the gastrointestinal tract is excreted in the urine. Elimination half time averages 28 ± 9 hours [4]. The US Department of Health and Human Services has determined that metallic nickel may be a carcinogen. The International Agency for Research on Cancer has concluded that some nickel compounds and metallic nickel are carcinogenic to humans. 

In Europe and North America, nickel sulphate is the most common sensitizer [5–8]. Although prevalence varies from one country to another, sensitization rates range from 9.2% in Germany to 14.3% in the United States, 15% in UAE, up to 19.1% in Turkey, and 19.9% in Singapore [9]. Overall, nickel sensitivity is 4 to 10 times more common in women than in men, which can be explained by the custom of girls piercing their ears at a very young age. Sensitivity begins between the ages of 2 and 5 years and increases to a peak at 10–15 years [10]. In North America, the frequency of positive patch-test reaction in children (age 0–18 years) was higher (28.3%) than that of adults (17.2%) [11]. 

Nickel-containing alloys are used in dental care, either in construction of restorations or as endodontic instruments and orthodontic appliances. Dental restorations leak nickel and chromium, another well-known allergenic compound. The oral manifestations of the contact allergy to nickel used in dentistry included lichen planus or stomatitis [12]. The blood concentration of nickel and chromium in patients with removable partial dentures made of nickel and chromium containing stainless steel showed a significant increased level [13, 14]. 

Stainless steel orthodontic materials and stainless steel crowns (SSC) are the two major devices in pediatric dentistry that contain nickel. Fixed orthodontic appliances release measurable amounts of nickel and chromium in the saliva and serum, without reaching toxic levels [14]. Nickel release from orthodontic appliances made from nickel-titanium and stainless steel increased over the first week after placement and then decreased over time [15]. The influence of chromium and nickel concentrations in saliva and their effects on gingival tissues during orthodontic treatment has been reported [16]. After 3 months, 20% of the females and 10% of the males showed an allergic reaction in a form of gingivitis, which disappeared a month after appliance removal. There is some evidence that oral contact with nickel and chromium may induce a state of partial tolerance to these materials in nonsensitized individuals, but the evidence is not significant statistically [17]. 

Prefabricated stainless steel crowns (SSCs) cemented to primary molars are the second most common dental device containing stainless steel used in children. Nickel sensitivity has been reported in children treated with old generation SSCs with high (up to 72%) nickel content [18]. Based on these findings, the new generation of SSC contains only 9%–12% nickel [19]. The aim of this study was to investigate the hypothesis that the new SSCs also release nickel, chromium, and iron that are absorbed by the roots of the primary molars on which the SSCs are cemented.

## 2. Material and Methods

The study group consisted of 17 primary molars covered with stainless steel crowns due to extensive loss of tooth material (SSC-ION 3M/ESPE co. St. Paul MN, USA) for at least 24 months and had 2 mm or more of mesial or distal root below the crown margins. The crowns were cemented with carboxylate cement (Durelon, 3M/ESPE AG, Seefeld, Germany). The teeth were collected after normal exfoliation (12 molars) or extraction performed due to orthodontic requests (5 molars). The control group consisted of 17 intact primary molars with at least 2 mm of mesial or distal root below the CEJ, normally exfoliated (14 molars) or extracted for orthodontic reasons (3 molars). 

**Figure 1:**
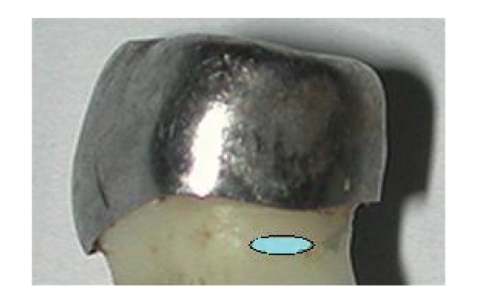
Location of chemical analysis on a SSC-covered primary molar, on the distal root surface.

The molars were kept dry in a plastic tube till the chemical examination was performed. An energy dispersive X-ray spectrometer (EDS) was used for chemical analysis. In order to enable a more accurate analysis of trace elements we had to work in a high-vacuum mode and high pressure. The teeth were coated with pure gold in order to keep the surface of the teeth intact in these conditions. The coating was of an inert material that did not react or interfere with the readings of nickel, chromium, and iron. The molars were inserted into the EDS chamber, parallel to the table. Chemical analysis was carried out on each tooth, on the outer aspect of mesial or distal root, 1 mm below the crown margins on the primary molars covered by SSC or 1 mm below the CEJ on control molars (Figure 1). The detection of elements was based on a program that analyzes the energy released from the elements on a standard area on each root. The basic tooth structure consists of hydroxyapatite (HA) crystals with the formula, [Ca_5_(PO_4_)_3_OH]_2_, and, as a result, the chemical composition of an intact tooth is primarily calcium, phosphate, oxygen, and carbon. For the current study, the elements which can leak from the stainless steel crowns to the root surface, that is, nickel, chromium, and iron, were included. 

All data were transferred to a computer and statistically analyzed using the SAS package. The Mann-Whitney *U* test was performed to determine the difference between values obtained between groups with  $\overset{´}{\alpha }=1\%$.

## 3. Results

The most prevalent elements found in the cementum of teeth covered by SSC and intact primary molars were calcium, phosphate, oxygen, and carbon with no significant differences between the primary molars covered with SSCs and intact primary molars. Traces of nickel, chromium, and iron were found in the cementum of the mesial or distal root in 23 out of 24 primary molars.  Table 1 summarizes the data on the main elements and traces elements found in the cementum of the mesial root of primary molars covered with SSCs and intact primary molars.

The concentrations (in mlwt%) of nickel, chromium, and iron found in the cementum of primary molars covered by SSCs were 5 to 6 times higher than the concentrations of the same elements in the cementum of intact primary molars. Nonparametric, Mann-Whitney *U* statistical analysis showed highly significant differences between the two groups (*P* < .0001) for the trace elements analyzed.

## 4. Discussion

The most commonly reported effect in individuals exposed to nickel is allergic contact dermatitis. After exposure to nickel, the endothelial cells that line the blood vessels produced immune-response-mediating molecules (cytokines) within 24 hours, suggesting that nickel itself was acting as the signal for T cells recruitment [20]. Nickel triggers an inflammatory response by directly activating human Toll-like receptor 4 (TLR4), and this activation was species specific [21]. The risk of developing an allergic reaction to nickel from stainless steel devices used as dental orthodontic appliances is well documented [22–26]. The reports are generally of patients with presensitization to nickel, that is, from earrings containing nickel. Both new and recycled brackets will corrode in the oral environment [27], and therefore, metal brackets should be made more resistant to corrosion and recycled brackets should not be used to avoid clinical side effects. In one study, [22] it was found that from 40 students without skin piercing, 4 out of 11 with a history of permanent braces had developed nickel allergy compared with none out of 22 without orthodontic treatment, suggesting the possibility of sensitization through dental devices. Oral exposure to nickel-containing metallic orthodontic appliances before sensitization to nickel (ear piercing) may have reduced the frequency of nickel hypersensitivity [28].

Toxicity and carcinogenicity of certain nickel compounds may be related to their uptake, transport, distribution, and retention at a cellular level [29]. 

The present study measured the absorption of metal ions released from SSC by the cementum of primary molars. Nickel is found normally in the saliva [30] and can be absorbed by intact teeth, but this level is significantly smaller when compared to the level detected after placement of fixed dental appliances containing nickel. A substantial amount of nickel, chromium, and iron was released from the stainless steel crowns and absorbed by the cementum of the root of primary molars below the crowns. The amount observed in molars covered with SSC was 5-6 times greater than the concentration in the cementum of intact primary molars and the differences were significant statistically. 

*In vivo* aged SSC surfaces showed significant morphologic alterations with wear and occlusal perforations as the most common findings. When the metal concentrations of new and *in vivo* aged SSC were compared, no statistically significant differences were found [31]. The quantitative analysis of the SSC components was analyzed as percentage of the component and not as the absolute amount. The authors stated that “The results of this study should not be viewed as a conclusive evidence of no compositional alteration of prefabricated pediatric metal crowns under clinical conditions” (p. 219). The release of metal components from the SSCs in the oral cavity will not affect the concentration of the elements in the SSCs. 

The present study showed that the amount of metallic ions absorbed by cementum was 0.4–1.5 mlwt% (Ni), 0.2–1.7 mlwt% (Cr), and 0.4–2.7 mlwt% (Fe). If this amount is reduced from the original content of the SSC (the low nickel crowns), Fe—72%, Cr—18%, and Ni—10%, it can be understood why the differences between new and *in vivo* aged SSC showed no significance statistically [31]. 

## 5. Conclusion

The chemical analysis showed that nickel, chromium, and iron were released from SSC and absorbed into the cementum of teeth covered with SSC. Since SSCs are also used to cover permanent molars, the influence of the release of these elements on the systemic health of children should be further investigated.

## Figures and Tables

**Table 1:** The quantity (in mlwt%) of Ni, Cr, and Fe in the cementum of the mesial root of primary molars.

		Ni			Cr			Fe	
	SSC	Intact	*P *	SSC	Intact	*P*	SSC	Intact	*P*
Mean	**0.86**	**0.16**	**<.0001**	**0.59**	**0.11**	**<.0001**	**1.13**	**0.20**	**<.0001**
*N*	17	17		17	17		17	17	
SD	0.42	0.10		0.37	0.07		0.77	0.13	
Min	0.40	0.00		0.20	0.00		0.40	0.00	
Max	1.50	0.30		1.70	0.20		2.70	0.40	

*Note*. SSC: molars covered with stainless steel crowns, Intact: intact molars, *P*: *P* value, *N*: number of molars examined, and SD: standard deviation.
